# Re-assessing *ZNF331* as a DNA methylation biomarker for colorectal cancer

**DOI:** 10.1186/s13148-018-0503-2

**Published:** 2018-05-29

**Authors:** Hege Marie Vedeld, Arild Nesbakken, Ragnhild A. Lothe, Guro E. Lind

**Affiliations:** 1 Department of Molecular Oncology, Institute for Cancer Research, Oslo University Hospital–Norwegian Radium Hospital, Oslo, Norway; 2 K.G. Jebsen Colorectal Cancer Research Centre, Oslo University Hospital, Oslo, Norway; 3 Department of Biosciences, Faculty of Mathematics and Natural Sciences, University of Oslo, Oslo, Norway; 4 Department of Gastrointestinal Surgery, Oslo University Hospital–Aker, Oslo, Norway; 5 Institute for Clinical Medicine, Faculty of Medicine, University of Oslo, Oslo, Norway

**Keywords:** Colorectal cancer, Diagnosis, DNA methylation, Prognosis, *ZNF331*

## Abstract

We have previously shown that aberrant promoter methylation of *ZNF331* is a potential biomarker for colorectal cancer detection with high sensitivity (71%) and specificity (98%). This finding was recently confirmed by others, and it was additionally suggested that promoter methylation of *ZNF331* was an independent prognostic biomarker for colorectal cancer (*n* = 146). In the current study, our initial colorectal cancer sample series was extended to include a total of 423 cancer tissue samples. Aberrant promoter methylation was found in 71% of the samples, thus repeatedly suggesting the biomarker potential of *ZNF331* for detection of colorectal cancer. Furthermore, multivariate Cox’s analysis indicated a trend towards inferior overall survival for colorectal cancer patients with aberrant methylation of *ZNF331*.

## Introduction

In cancer, increased promoter DNA methylation is a frequent event commonly occurring early in tumor development. Methylated DNA sequences may serve as tumor biomarkers in liquid biopsies for detecting cancer and for predicting patient prognosis [1].

In 2011, we filed a patent application covering methylation of *ZNF331* (Zinc finger protein 331) as a biomarker for gastrointestinal cancers [2]. *ZNF331* was shown by Yu et al. to be inactivated by promoter methylation in gastric cancer, providing the cancer cells with increased growth potential and invasiveness [3]. We also found a high methylation frequency in patients with gastric cancer (80%) and to a lesser extent in patients with pancreatic cancer (40%) and cholangiocarcinomas (26%) [4]. Most importantly, we reported high sensitivity (71%) and specificity (98%) for *ZNF331* methylation in colorectal cancer early 2015, strengthening the potential of *ZNF331* as a biomarker for colorectal cancer detection [4]. Interestingly, these findings were recently confirmed, further supporting the biomarker potential of *ZNF331* in colorectal cancer [5]. The same study also suggested aberrant promoter methylation of *ZNF331* as an independent prognostic marker for colorectal cancer, analyzing 146 samples [5]. In the present study, we analyzed the effect of *ZNF331* methylation on overall survival, including altogether 423 colorectal tissue samples.

## Results and discussion

Methylation of the *ZNF331* promoter was found in 71% (301/423) of the patients with colorectal cancer and was associated with localization in the right colon, microsatellite instability (MSI), and the *BRAF*^*V600E*^ mutation. Furthermore, *ZNF331* methylation was strongly associated with CpG island methylator phenotype (CIMP) and *MLH1* methylation (Table 1). Wang et al. [5] reported a similar methylation frequency of *ZNF331* in colorectal cancer (67%; 98/146). However, in contrast to our data Wang et al. did not find associations between methylated *ZNF331* and *BRAF* mutation, CIMP nor *MLH1* methylation, which may be explained by differences in sample size (Wang et al., *n* = 146; current study, *n* = 423), marker panels to define CIMP, method to identify methylation, age (median age Wang et al. 60; current study 72), and/or ethnicity (Wang et al.: Asian; current study: Caucasian).

**Table 1:** Associations between *ZNF331* methylation and clinical and molecular features

	Total	ZNF331 unmethylated	ZNF331 methylated	*P* value
	*n*	*n* (%)	*n* (%)	
No. of patients	423	122 (29)	301 (71)	
Gender				0.165
Male	213	68 (32)	145 (68)	
Female	210	54 (26)	156 (74)	
Age				0.074
< 60	70	26 (37)	44 (63)	
60–74	178	55 (31)	123 (69)	
≥ 75	175	41 (23)	134 (77)	
Stage				0.683
I	79	20 (25)	59 (75)	
II	169	51 (30)	118 (70)	
III	118	32 (27)	86 (73)	
IV	56	19 (34)	37 (66)	
Localization				< 0.001
Right colon	167	27 (16)	140 (84)	
Left colon	130	47 (36)	83 (64)	
Rectum	121	46 (38)	75 (62)	
MSI status				< 0.001
MSS	325	111 (34)	214 (66)	
MSI	89	8 (9)	81 (91)	
*BRAF*				< 0.001
*BRAF* wt	356	120 (34)	236 (66)	
*BRAF* mut	67	2 (3)	65 (97)	
*CIMP*				< 0.001
*CIMP−*	355	121 (34%)	234 (66)	
*CIMP+*	65	0 (0)	65 (100)	
*MLH1* methylation				< 0.001
*MLH1* unmeth	360	117 (32.5)	243 (67.5)	
*MLH1* meth	60	4 (7)	56 (93)	
Series				0.439
Oslo 3	59	14 (24)	45 (76)	
Oslo 2	364	108 (30)	256 (70)	

*Meth* methylated, *mut* mutation, *No.* number, *unmeth* unmethylated, *wt* wild type

**Fig. 1:**
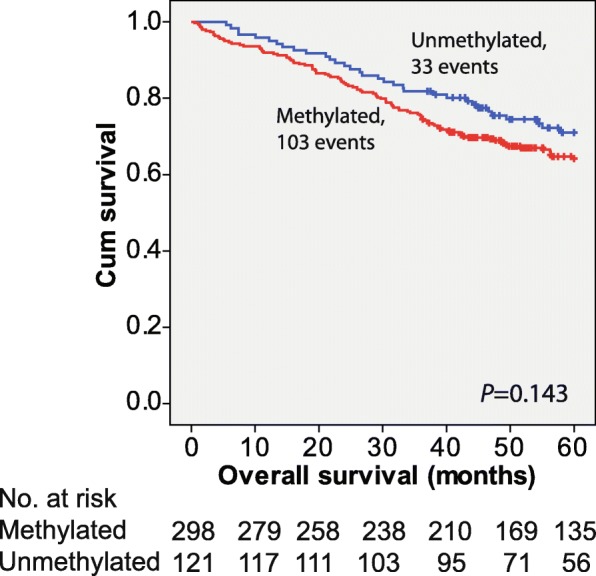
Effect of *ZNF331* promoter methylation on overall survival modeled by the Kaplan-Meier method and compared using the log-rank test

Wang et al. [5] further reported that patients with *ZNF331* promoter methylation had a worse prognosis than patients with unmethylated promoters. Our results were in accordance with their study, although statistical significance was not reached in the multivariate Cox regression model adjusting for age and stage (HR = 1.44 (0.97–2.14), *P* = 0.069; Table 2). The univariate model is presented in Fig. 1 (*P* = 0.143).

**Table 2:** Multivariate Cox proportional hazard analysis with overall survival as endpoint

	Patients, *n*	Multivariate HR (95% CI)	*P* value
Age			
< 60	70	1.00 (ref)	
60–74	176	1.70 (0.91–3.18)	0.099
≥ 75	173	3.42 (1.84–6.34)	< 0.001
Stage			
I	78	1.00 (ref)	
II	168	1.24 (0.66–2.34)	0.498
III	117	2.32 (1.24–4.34)	0.009
IV	56	11.10 (5.91–20.85)	< 0.001
*ZNF331* methylation			
*ZNF331* unmeth	121	1.00 (ref)	
*ZNF331* meth	298	1.44 (0.97–2.14)	0.069

Variables not selected by the backward likelihood method to be included in the final model: series, gender, CIMP-, MSI-, and *BRAF* mutation status

*Meth* methylated, *unmeth* unmethylated

In conclusion, in an extended series of colorectal cancer samples, we have showed the potential of promoter methylation of *ZNF331* as a biomarker for colorectal cancer detection. We have further provided data indicating a trend towards poorer prognosis for patients with *ZNF331* methylation.

## Material and methods

### Colorectal cancer tissue samples

This study included 423 colorectal cancer tissue samples. Fifty-nine of the samples were obtained from several different hospitals in the southeast region of Norway in the period 1987–1989 (Oslo 3 series; described in [6]), and 364 of the samples were obtained from patients undergoing surgical resection at the Oslo University Hospital–Aker from 2005 to 2011 (Oslo 2 series; described in [7, 8]). Survival data was available for 419 patients (Oslo 3, *n* = 59; Oslo 2, *n* = 360).

### Bisulfite treatment and quantitative methylation-specific PCR (qMSP)

DNA from cancer tissue samples were bisulfite treated using the EpiTect Bisulfite Kit (Qiagen), and the samples were purified using the QIAcube (Qiagen).

Quantitative methylation-specific PCR (qMSP) was used to analyze the methylation of the *ZNF331* promoter (NM_018555), with primers and probe sequences as reported earlier [4]. The method was performed as previously described [4, 9], with the ALU-C4 element as a normalization control [10]. As described in ref. [4], samples with percent methylated reference (PMR) values ≥ 1 were considered methylated. Information about MSI, CIMP, *MLH1* methylation, and *BRAF* mutation status were available from previous studies [11, 12].

### Statistical analyses

Associations between *ZNF331* methylation and clinicopathological data were analyzed by Pearson chi-square or Fisher’s exact tests. For all analyses, patients were divided into three age groups (< 60 years, 60–74 years, and ≥ 75 years). Breakpoints were chosen as previously described [11]. Overall survival was used as endpoint in the survival analyses and was calculated from time of surgery until death of any cause. Cases were censored at last follow-up. The univariate effect of *ZNF331* on survival was modeled by the Kaplan-Meier method and compared using the log-rank test. A multivariate Cox’s proportional hazard model was generated by a stepwise selection procedure (backward likelihood model) in order to identify a subset of relevant predictor variables from the set of available clinicopathological data (series, age, stage, gender, CIMP-, MSI-, *BRAF-*, and *ZNF331* methylation status). Hazard ratios (HRs) and 95% confidence intervals (CIs) were derived from the model, and significance of the parameters was assessed using Wald’s test. To evaluate the assumption of proportionality, a chi-square test was performed. A *P* value < 0.05 was considered statistically significant. The analyses were performed using IBM SPSS Statistics 21 and R version 3.4.1.

## Abbreviations

CIMP: CpG island methylator phenotype
